# Global profiling of secreted protease activities in *Coccidioides posadasii*

**DOI:** 10.1016/j.crmicr.2026.100671

**Published:** 2026-10-17

**Authors:** Ana K. Serrano, Tahirah Williams, Austin M. Perry, Mélanie A.C. Ikeh, Tvisha Devavarapu, Pavla Fajtova, Zhenze Jiang, Clarissa J. Nobile, Anthony J. O’Donoghue

**Affiliations:** a Center for Discovery and Innovation in Parasitic Diseases, Skaggs School of Pharmacy and Pharmaceutical Sciences, University of California San Diego, La Jolla, CA, USA; b Department of Molecular and Cell Biology, University of California Merced, Merced, CA, USA; c Quantitative and Systems Biology Graduate Program, University of California Merced, Merced, CA, USA; d Health Sciences Research Institute, University of California Merced, Merced, CA, USA; e Center for Cellular and Biomolecular Machines, University of California Merced, Merced, CA, USA

**Keywords:** Valley fever, Secreted proteases, Coccidioidomycosis, *Coccidioides*

## Abstract

Coccidioidomycosis (Valley fever), caused by inhalation of *Coccidioides* arthroconidia, remains a diagnostic challenge due to its clinical similarity to other respiratory illnesses and the absence of a rapid, definitive diagnostic test. Current approaches, including serology and imaging, often result in delayed or inconclusive diagnosis. Here, we profiled secreted protease activity in cell-free conditioned media from a single strain of *Coccidioides posadasii*. This activity could potentially form the basis of a protease-targeted diagnostic assay for clinical specimens. Using multiplex substrate profiling by mass spectrometry (MSP-MS) applied to cell-free conditioned media, we characterized global proteolytic activity at acidic (pH 2.5) and neutral (pH 7.4) conditions. At acidic pH, we detected dominant aspartyl protease activity that was inhibited by pepstatin and exhibited substrate preferences similar to human cathepsin D. Consistent with these findings, a commercial cathepsin D fluorogenic substrate was efficiently cleaved under acidic conditions. At neutral pH, we observed distinct proteolytic activity consistent with contributions from both exopeptidases and endopeptidases. Inhibition profiling and fluorogenic substrate assays further suggested involvement of serine and threonine proteases. These findings provide an activity-based characterization of secreted proteases from a single *C. posadasii* strain and establish a resource for future studies evaluating their biological and diagnostic relevance.

## Highlights

• Current approaches to detecting coccidioidomycosis (Valley fever) result in delayed or inconclusive diagnosis.
• We investigate secreted protease activity from *Coccidioides posadasii* as a basis for developing a protease-targeted diagnostic assay for clinical specimens.
• We detected robust protease activity in conditioned media when assayed at both acid and neutral pH.
• Inhibition profiling and fluorogenic substrate assays revealed the involvement of aspartyl, serine and threonine proteases.

## Graphical abstract

**Figure:**
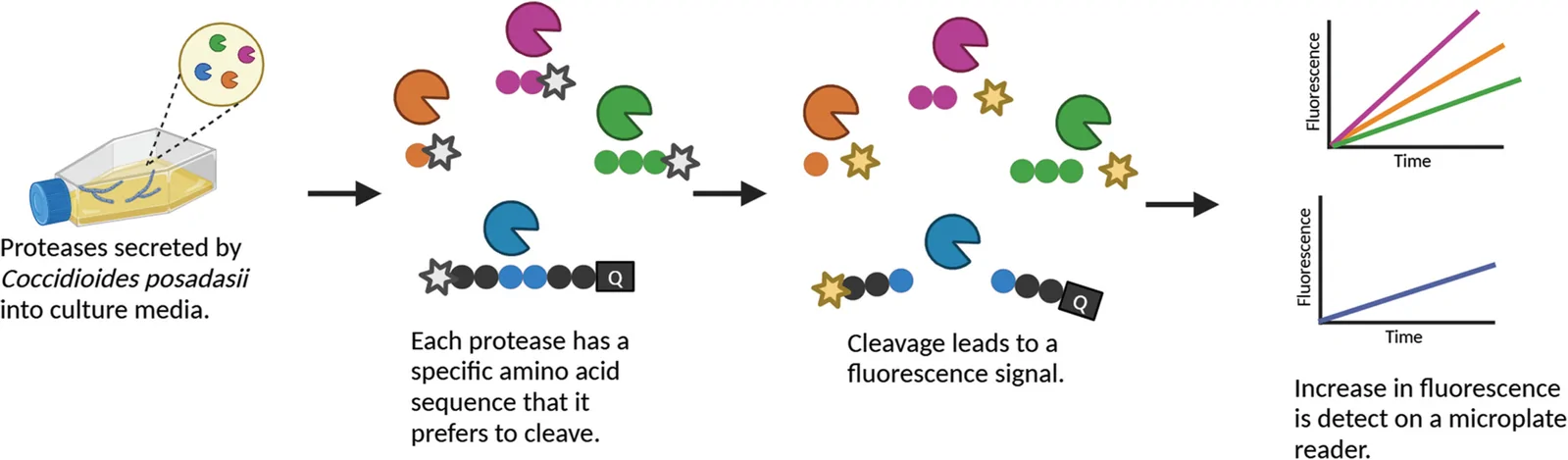

## Introduction

Coccidioidomycosis, commonly known as Valley fever, is an infection caused by inhalation of arthroconidia from the pathogenic fungi *Coccidioides immitis* and *Coccidioides posadasii* (Johnson et al., 2014; Morales et al., 2026; McHardy et al., 2023) . These species are endemic to arid and semiarid regions of the western and southwestern United States, Mexico, and parts of Central and South America (Johnson et al., 2014; Jackson et al., 2024; Williams and Chiller, 2022). Although their geographic ranges overlap, *C. immitis* is concentrated primarily in California and Washington, whereas *C. posadasii* is more broadly distributed across Arizona, Nevada, New Mexico, Texas, Mexico, and Central and South America (Kirkland and Fierer, 2018). Approximately 20,000 cases of coccidioidomycosis are reported annually in the United States, although the true burden is estimated to be substantially greater because of underdiagnosis, misdiagnosis, incomplete reporting, and failure to seek medical care (Williams et al., 2025; Centers for Disease Control and Prevention, 2024).

*Coccidioides* species inhabit soil, where periods of increased moisture can promote mycelial growth and subsequent hot and dry conditions favor formation and dispersal of infectious arthroconidia (Head et al., 2022; Jackson et al., 2024). Infection occurs when airborne arthroconidia are inhaled, particularly following wind or activities that disturb contaminated soil (Head et al., 2022; Freedman et al., 2018). In the lungs, arthroconidia undergo a morphological transition to the parasitic spherule phase, and infection can produce respiratory symptoms including cough, fever, chest pain, and fatigue (Jackson et al., 2024). Although most infections remain pulmonary, a small proportion disseminate beyond the lungs and can cause severe or life-threatening disease (Jackson et al., 2024). Risk of exposure is increased among individuals who live or work in endemic regions and experience frequent contact with airborne dust, including agricultural and construction workers and incarcerated populations (Freedman et al., 2018; McCurdy et al., 2020; Wheeler et al., 2015; Blow, 2023).

Valley fever remains diagnostically challenging because its clinical presentation frequently overlaps with other respiratory illnesses, particularly community-acquired pneumonia (McHardy et al., 2023; Crum, 2022). Diagnosis therefore relies on a combination of clinical and epidemiologic assessment, serological testing, culture or direct detection of the organism, and radiographic findings (McHardy et al., 2023). Serological assays are widely used but can yield negative results early in infection before detectable antibody responses have developed, whereas fungal culture can require several days and must be performed with appropriate biosafety precautions (McHardy et al., 2023). Radiographic abnormalities can support a diagnosis but are not specific for coccidioidomycosis and may resemble other pulmonary diseases (McHardy et al., 2023; Peçanha-Pietrobom et al., 2023). Consequently, diagnosis is frequently delayed or initially missed. Enhanced surveillance studies have reported a median interval of 38 days between seeking medical care and diagnosis, with approximately 70% of patients receiving an alternative diagnosis before being tested for coccidioidomycosis (Blow, 2023). More recent national analyses likewise demonstrate that diagnostic delays remain common (Barber et al., 2026). These limitations underscore the need for rapid, sensitive, and pathogen-directed diagnostic approaches that can detect infection early in the course of disease.

Fungi secrete diverse hydrolytic enzymes that facilitate nutrient acquisition and adaptation to their environments. In pathogenic fungi, extracellular proteases can additionally contribute to virulence by degrading host structural proteins, disrupting tissue barriers, and modulating host immune responses (Girard et al., 2013; Shah et al., 2013; Yang et al., 2007). Fungal proteases can degrade host proteins including collagen and keratin (Gradišar et al., 2005; Wanderley MC de et al., 2016) and can influence inflammatory signaling, including cytokine production and activation of the NLRP3 inflammasome (Pietrella et al., 2013). These properties make secreted proteases attractive candidates both for understanding fungal pathogenesis and as potential activity-based biomarkers of infection.

A previous quantitative mass spectrometry study aimed at identifying fungal protein biomarkers in lung tissue from patients with Valley fever detected multiple *Coccidioides* proteases, including ten enzymes listed in Table 1 (Mitchell et al., 2020). Among these, the aspartyl protease Pep1 has been biochemically and immunologically characterized. Pep1 is expressed during infection, elicits antibody responses in patients, and immunization with recombinant Pep1 reduces fungal burden in a murine model of coccidioidomycosis (Tarcha et al., 2006). Despite these findings, the enzymatic activities, substrate specificities, and broader biochemical properties of most *Coccidioides* proteases detected during infection remain poorly defined. This gap limits our understanding of their potential contributions to fungal physiology and pathogenesis and their utility as activity-based biomarkers.

**Table 1:** *C. posadasii* proteases previously identified from studies of Valley fever patients.

Uniprot ID	Protease Name	Protease Family	References
**A0A0J6F9D9**	Xaa-Pro aminopeptidase	Metallo	Mitchell et al., 2020
**A0A0J6FJ97**	Metallo aminopeptidase	Metallo	Mitchell et al., 2020
**A0A0J6FEL7**	Aspartyl aminopeptidase	Metallo	Mitchell et al., 2020
**Q3S565**	Pep1	Aspartyl	Tarcha et al., 2006
**Q3HYC2**	Pep2	Aspartyl	Mitchell et al., 2020
**A0A0J6FSX0**	MetAP2	Metallo	Mitchell et al., 2020
**Q0MYP0**	MPP	Metallo	Mitchell et al., 2020
**A0A0J6F3G1**	Proteasome	Threonine	Mitchell et al., 2020
**A0A0J6FFQ8**	Subtilisin	Serine	Mitchell et al., 2020
**A0A0J6F7Q7**	Thimet oligopeptidase	Metallo	Mitchell et al., 2020
**C5P212**	Carboxypeptidase Y	Serine	Tarcha et al., 2006
**C5P3X6**	Mep1	Metallo	Hung et al., 2005

Proteases are attractive candidates for activity-based diagnostics because their catalytic activity provides inherent signal amplification, with a single enzyme capable of cleaving many substrate molecules. This enables sensitive detection using simple fluorogenic or colorimetric reporters without the need for additional amplification steps. In principle, such assays can generate a direct activity-based readout within minutes to hours, rather than relying on development of a host antibody response or prolonged culture. Specificity can also be engineered into the reporter substrate by optimizing the peptide sequence for cleavage by the target protease while minimizing recognition by host enzymes. If validated in clinical specimens, these features could provide rapid, pathogen-directed evidence of infection that complements existing serological and culture-based approaches. In previous work, we demonstrated that disease-associated proteolytic signatures can be leveraged for diagnostic development. In pancreatic cyst fluid, we identified a protease activity that distinguished premalignant from benign cysts and developed an optimized fluorogenic substrate that achieved 100% specificity and 93% sensitivity (Ivry et al., 2017). In infectious disease applications, we have also identified two secreted proteases from the human fungal pathogen *Candida albicans* that could serve as biomarkers of biofilm formation (Winter et al., 2016). These studies support the feasibility of using pathogen-associated protease activity as a basis for diagnostic assay development.

Here, we profiled protease activity in cell-free conditioned media from a single strain of *C. posadasii* using multiplex substrate profiling by mass spectrometry (MSP-MS) under acidic and neutral pH conditions. We identified distinct proteolytic activities attributable to multiple protease classes and validated selected activities using fluorogenic reporter substrates and protease inhibitors. Several of the activities were consistent with protease classes previously detected in *Coccidioides*-infected lung tissue (Fig. 1). These studies provide an activity-based framework for investigating the extracellular protease repertoire of *C. posadasii* and for future evaluation of protease-targeted diagnostic approaches for Valley fever.

**Fig. 1:**
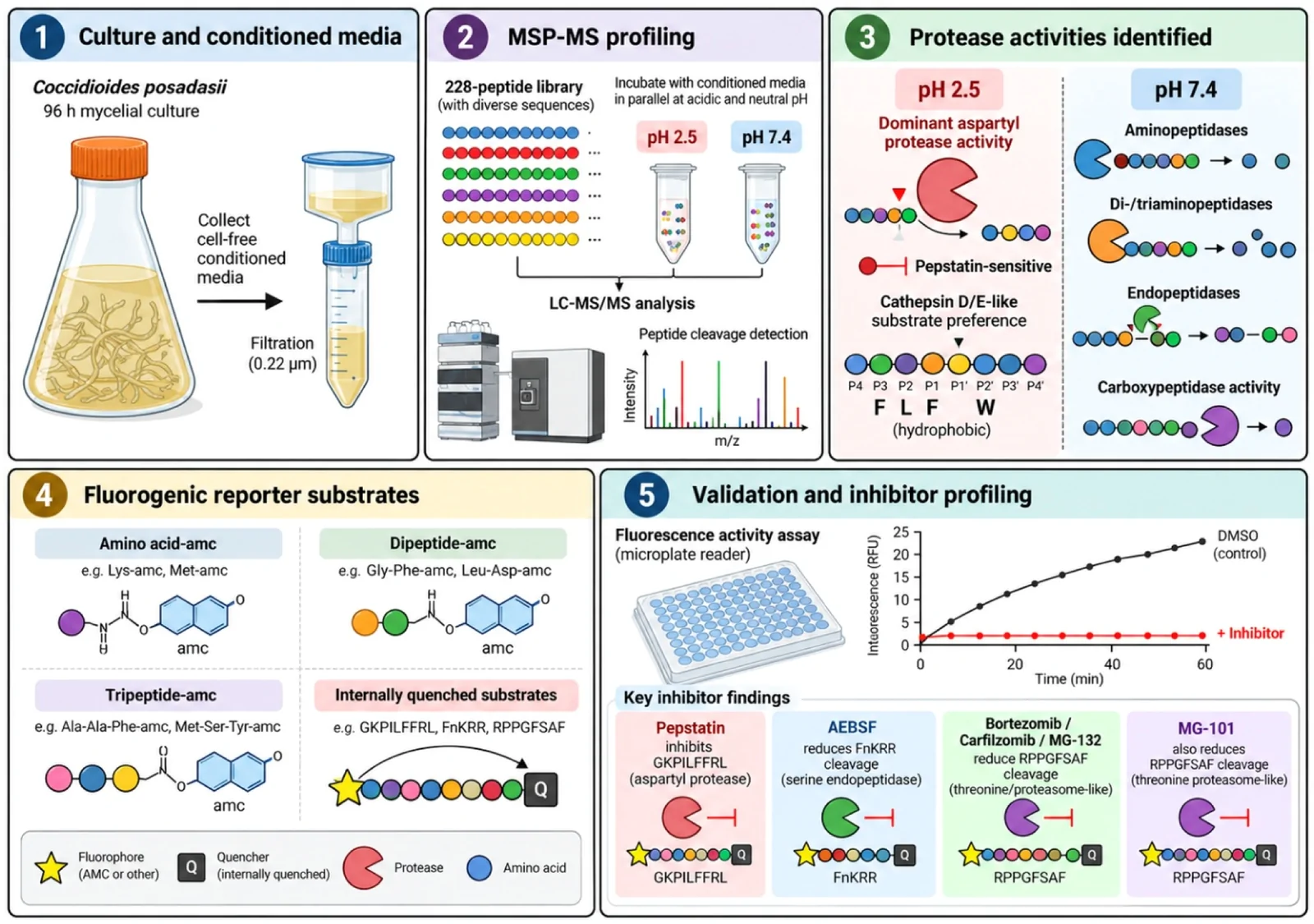
Overview of the experimental strategy used to profile secreted protease activities of *Coccidioides posadasii. C. posadasii* was grown for 96 h under mycelial growth conditions, and cell-free conditioned media was collected by filtration. Secreted protease activity was profiled using multiplex substrate profiling by mass spectrometry (MSP-MS) with a library of 228 synthetic peptides under acidic (pH 2.5) and neutral (pH 7.4) conditions. At pH 2.5, the predominant activity was pepstatin-sensitive and consistent with an aspartyl protease exhibiting a cathepsin D/E-like substrate preference. At pH 7.4, MSP-MS revealed more extensive proteolytic activity, including aminopeptidase, di- and triaminopeptidase, endopeptidase, and potential carboxypeptidase activities. Selected activities were subsequently evaluated using fluorogenic reporter substrates, including amino acid-, dipeptide-, and tripeptide-amc substrates and internally quenched peptide substrates. Protease inhibitor profiling was used to further classify selected activities. Pepstatin inhibited cleavage of the GKPILFFRL substrate, supporting aspartyl protease activity; AEBSF reduced cleavage of FnKRR, consistent with serine protease activity; and bortezomib, carfilzomib, MG-132, and MG-101 reduced cleavage of RPPGFSAF, supporting threonine/proteasome-like proteolytic activity. These approaches enabled activity-based profiling and biochemical classification of multiple protease activities present in *C. posadasii* conditioned media. amc, 7-amino-4-methylcoumarin; MSP-MS, multiplex substrate profiling by mass spectrometry. Created with BioRender.com.

## Materials and methods

### Culture conditions and media preparation

*C. posadasii* strain NR-166 (*Δcts2/Δard1/Δcts3*) was used for all assays. Arthroconidia from NR-166 were harvested from 6-week-old 2X concentrated glucose yeast extract (2X GYE) agar plates following established protocols (Mead et al., 2020). An inoculum of 1 × 10⁶ arthroconidia was introduced into a 125 mL baffled Erlenmeyer flask containing 50 mL of 2X GYE medium. The culture was incubated at 30°C with agitation at 150 rpm for 96 h, after which the majority of cells were in the mycelial form. After incubation, fungal cells were separated from the conditioned media by filtration through a sterile 40 µm nylon mesh cell strainer. The resulting conditioned media was further filtered with a 0.22 µm syringe filter and stored at -80°C.

Cell-free conditioned media was concentrated and buffer exchanged into Dulbecco's phosphate buffered saline pH 7.4 (DPBS) using 10 kDa centrifugal filters (Millipore #UFC901008) and centrifuged at 4000 x g at 4°C. Protein levels were quantified using the bicinchoninic acid assay (Pierce #23225). Samples were aliquoted and stored at -80°C.

### **Multiplex substrate profiling by mass spectrometry** (**MSP-MS**)

Proteolytic activity in *C. posadasii* conditioned media was analyzed using a library of 228 synthetic tetradecapeptides designed to encompass known protease cleavage sites (Lapek et al., 2019; O’Donoghue et al., 2012; Rohweder et al., 2023). Assays were performed in quadruplicate using the 228-peptide library at a final concentration of 0.5 μM for each peptide and *C. posadasii* protein at a final concentration of 20 μg/mL. Reactions were incubated at room temperature for 1 hour in either 20 mM citrate phosphate buffer, pH 2.5, or DPBS, pH 7.4. At 15 minutes and 1 hour, 20 μL aliquots were removed and quenched by the addition of guanidine hydrochloride (GuHCl) (MP Biomedicals) to a final concentration of 6.4 M, followed by immediate storage at −80°C. Control reactions consisted of *C. posadasii* protein pre-incubated with 6.4 M GuHCl to inactivate the enzyme before the addition of the peptide library. All samples were desalted using C18 solid-phase extraction and dried by vacuum centrifugation.

Cleavage products were identified and quantified by nano-LC–MS/MS. Briefly, 0.4 μg of peptides was injected into a Q-Exactive mass spectrometer (Thermo Fisher Scientific) coupled to an Ultimate 3000 High-Performance Liquid Chromatography (HPLC) system (Thermo Fisher Scientific). Peptides were separated by reversed-phase chromatography on a C18 column (1.7 μm bead size, 75 μm × 25 cm, 65°C) at a flow rate of 300 nL/min using a 60-minute linear gradient from 5% to 30% solvent B (0.1% formic acid in acetonitrile) in solvent A (0.1% formic acid in water). Survey scans were acquired over an *m/z* range of 150–2000 (70,000 resolution at *m/z* 200, Automatic Gain Control (AGC) target 3 × 10^6^, 100 ms maximum injection time). Data-dependent acquisition (DDA) was used for MS/MS analysis, with higher-energy collisional dissociation (HCD) fragmentation (28 normalized collision energy) performed on the 12 most intense precursor ions (17,500 resolution at *m/z* 200, AGC target 1 × 10^5^, 50 ms maximum injection time, 20 s dynamic exclusion).

Data were processed using PEAKS 8.5 software (Bioinformatics Solutions Inc.). MS/MS data were searched against the tetradecapeptide library sequences and corresponding decoy sequences (reverse order). Precursor and fragment mass tolerances were set to 20 parts per million and 0.01 Da, respectively. No enzyme specificity was specified during the database search. Peptide identifications were filtered to a 1% false discovery rate using the target-decoy approach. Label-free quantification was performed, and data were median-normalized and filtered using a peptide quality threshold of 0.3. Missing and zero values were imputed using randomly generated numbers from a normal distribution defined by the mean and standard deviation of the lowest 5% of the data. Cleaved peptides were defined as increasing at 8-fold or more between the control reaction and the 1-hour reaction with q value <0.05. Processed mass spectrometry data can be found in Supplementary File 1 and raw files can be retrieved from the MassIVE server (https://massive.ucsd.edu/ProteoSAFe/static/massive.jsp) using the identifier MSV000102535 or from Proteome Central (https://www.proteomexchange.org/) using the identifier PXD081341.

### Fluorescent substrate assays

Fluorescence assays were performed using 20 μg/mL of *C. posadasii* protein and 2.5 μM of substrate at room temperature in a Synergy HTX microplate reader (BioTek, VT, USA). Assays were performed on 384 well low volume plates in a final volume of 10 μL. All assays were performed in three technical replicates. For substrates containing a C-terminal 7-amino-4-methylcoumarin (amc) group, the excitation and emission wavelength were 360 nm and 460 nm, respectively. For internally quenched substrates containing an N-terminal 4-methylcoumarin amide (mca) group and C-terminal lysine-2,4-dinitrophenol (Lys(dnp)) group, the excitation and emission wavelengths were 320 nm and 400 nm, respectively.

The following peptide substrates were purchased from vendor catalogs: Asp-amc, Arg-amc, Trp-amc, Gly-Phe-amc, Gly-Arg-amc, Z-Arg-Arg-amc and Ala-Ala-Phe (Bachem); Met-amc, Lys-amc, Gly-Pro-amc, Leu-Asp-amc (Peptides International); Leu-amc (Abcam); Arg-Arg-amc (Santa Cruz Biotechnology); Ser-amc (Sigma); Nle-amc, Gly-Nle-amc, mca-Gly-Lys-Pro-Ile-Leu-Phe-Phe-Arg-Leu-Lys(dnp)-DArg-NH2, mca-Ser-Glu-Val-Asn-Leu-Asp-Ala-Glu-Phe-Lys(dnp)-OH, mca-Asp-Glu-Val-Asp-Ala-Pro-Lys(dnp)-OH, mca-Tyr-Val-Ala-Asp-Ala-Pro-Lys(dnp)-OH, and mca-Arg-Pro-Pro-Gly-Phe-Ser-Ala-Phe-Lys(dnp)-OH (CPC Scientific); Succinyl-Ala-Ala-Phe-amc (Echelon Biosciences Inc). The following substrates were custom synthesized by GenScript: Gly-Ile-Gln-amc, Lys-Trp-Leu-amc, Met-Ser-Tyr-amc, Asn-Phe-Nle-amc, Arg-Pro-Val-amc, Thr-His-Ala-amc, mca-Phe-Nle-Lys-Arg-Arg-Lys(dnp)-OH, mca-Phe-Val-Thr-Lys-Gly-Lys(dnp)-OH, mca-Phe-Leu-Val-Arg-Gly-Lys(dnp)-OH and mca-Phe-Arg-Ile-His-Gly-Lys(dnp)-OH.

Assays were performed in 0.1 M citrate-phosphate buffer for pH 2.0–7.0, 0.5 M Tris-HCl buffer for pH 7.0–9.5 or PBS pH 7.4. All assay buffers contained 1 mM dithiothreitol (DTT). For studies conducted across a range of pH values, activity in relative fluorescence units per second (RFU/s) was normalized to 0.1 M citrate-phosphate pH 7.0 and 0.5 M Tris-HCl buffer for pH 7.0–9.5.

### Inhibitor screening

For inhibitor screening studies 100 μg/mL of *C. posadasii* protein was used for the 4-(2-aminoethyl)benzenesulfonyl fluoride hydrochloride (AEBSF), tosyl phenylalanyl chloromethyl ketone (TPCK), tosyl-L-lysyl-chloromethane hydrochloride (TLCK), nafamostat, bortezomib, carfilzomib, and E-64 assays, while 20 μg/mL of *C. posadasii* protein was used for MG-132, MG-101, and pepstatin assays. The protein was preincubated in assay buffer for 30 min, followed by a 10-min incubation with inhibitor prior to substrate addition. Substrates were tested at a final concentration of 2.5 μM. Inhibitors were used at final concentrations of 1 mM AEBSF; 10 μM TPCK, TLCK, nafamostat, bortezomib, carfilzomib, E-64, and pepstatin; and 100 μM MG-132 and MG-101. All assays included negative controls with no inhibitor (DMSO vehicle control). Assays were conducted in triplicate wells in PBS containing 0.01% Tween and 1 mM DTT, pH 7.4, in a total volume of 10 μL. Readings were taken using a BioTek HTX multimode plate reader (λex = 320 nm, λem = 400 nm). The protease inhibitors used in this study, their primary target classes, and their observed effects on substrate cleavage are summarized in Table 2.

**Table 2:** Protease inhibitors used in this study, their primary target classes, and effects on *C. posadasii* substrate cleavage.

Inhibitor	Primary/Commonly Used Target	Observed Effect
Pepstatin	Aspartyl proteases	Inhibited GKPILFFRL cleavage at pH 3.5 & 5.5
AEBSF	Serine proteases	Reduced FnKRR cleavage at pH 7.4
TPCK	Serine proteases	No significant inhibition
TLCK	Serine proteases	No significant inhibition
Nafamostat	Serine proteases	No significant inhibition
Bortezomib	Proteasome/threonine protease activity	Reduced RPPGFSAF cleavage at pH 7.4
Carfilzomib	Proteasome/threonine protease activity	Reduced RPPGFSAF cleavage at pH 7.4
MG-132	Proteasome inhibitor; can have off-target effects	Reduced RPPGFSAF cleavage at pH 7.4
MG-101	Cysteine protease inhibitor; also reported to inhibit proteasome activity	Reduced RPPGFSAF cleavage at pH 7.4
E-64	Cysteine proteases	No significant inhibition

## Results

### Acidic pH profiling identifies a dominant aspartyl protease activity in *C. posadasii* conditioned media

Previous studies identified the aspartyl protease Pep1 as a prominent component of the parasitic cell wall of *C. posadasii* (Tarcha et al., 2006). Because fungal aspartyl proteases are typically active under acidic conditions (Mamo and Assefa, 2018; O’Donoghue et al., 2008), we first characterized protease activity in *C. posadasii* cell-free conditioned media at pH 2.5.

Conditioned media was incubated with a library of 228 rationally designed 14-mer peptides and analyzed by multiplex substrate profiling by mass spectrometry (MSP-MS). 18 cleavage products were detected. 17 cleavage sites occurred between residues 3 and 12 of the peptides, consistent with endoprotease activity, whereas a single cleavage event occurred between residues 13 and 14, suggesting the presence of a carboxypeptidase (Fig. 2A). Pre-incubation of conditioned media with the broad-spectrum aspartyl protease inhibitor pepstatin abolished 16 of the 18 cleavage events. For example, the peptide FIVFILWRTEHHAn was cleaved between Phe and Ile, resulting in time-dependent accumulation of the product ILWRTEHHAn in vehicle-treated samples, whereas no product formation was observed in the presence of pepstatin (Fig. 2B). In contrast, the putative carboxypeptidase cleavage and one endoprotease cleavage site remained pepstatin-insensitive, as illustrated by cleavage of IPTNIRASVPQWLW between Pro and Gln (Fig. 2C).

**Fig. 2:**
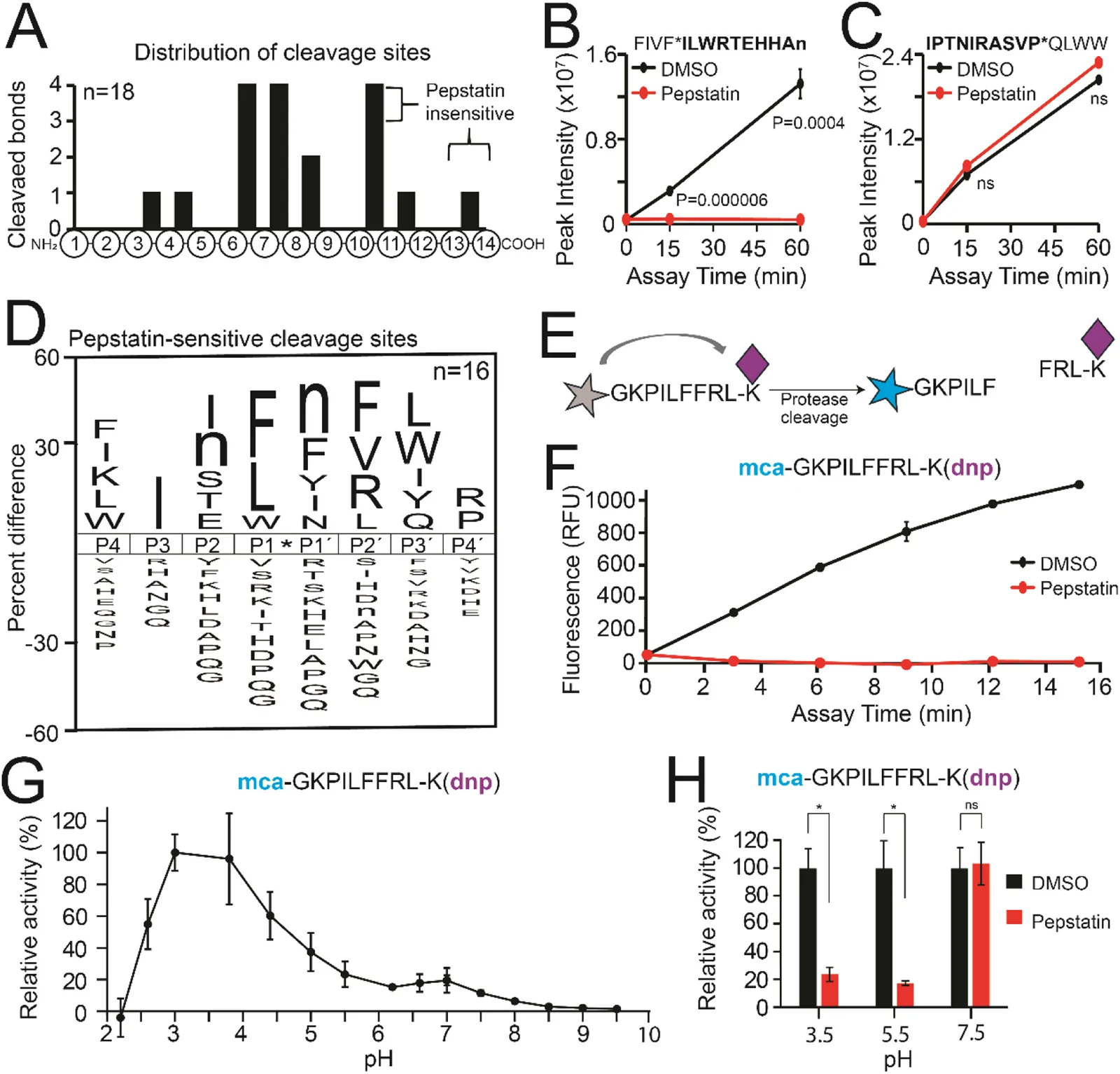
Substrate specificity profiling of *C. posadasii* secreted proteases at pH 2.5. (**A**) Distribution of cleavage sites within the 14-mer peptide library. (**B**) Representative mass spectrometry data showing cleavage of a peptide substrate by *C. posadasii* proteases in the absence (DMSO vehicle control) but not the presence of pepstatin. Peak intensity was determined by label-free quantification. (**C**) Representative mass spectrometry data showing cleavage of a peptide substrate by *C. posadasii* proteases that is unaffected by pepstatin. (**D**) Substrate signature (P4 to P4′) of the aspartyl protease, generated using iceLogo. Amino acids above the midline are enriched, while those below are not frequently found. The height of each amino acid represents the percent difference between the observed frequency and the background frequency. Lowercase n corresponds to norleucine. (**E**) Schematic representation of the internally quenched fluorescent substrate. (**F**) Quantification of *C. posadasii* acid protease activity over time at pH 2.5 in the absence (DMSO vehicle control) and presence of pepstatin. Fluorescence is expressed in relative fluorescence units (RFU). (**G**) Substrate cleavage rates (RFU/s) at various pH values. Cleavage rates were normalized to the highest observed activity. (**H**) Effect of pepstatin on protease activity at pH 3.5 (optimal), 5.5, and 7.5. MSP-MS assays were performed in quadruplicate technical replicates, and fluorogenic assays were performed in triplicate. Statistical significance was determined using a paired t-test with * corresponding to p < 0.05.

To define substrate preferences of the pepstatin-sensitive activity, we generated a substrate signature using iceLogo software (Colaert et al., 2009) using the P4 to P4′ residues surrounding the 17 cleavage sites that were sensitive to pepstatin. This dominant acidic protease activity preferentially cleaved substrates containing Ile at P3; Ile and norleucine (Nle) at P2; Phe and Leu at P1; Nle and Phe at P1′; and Phe, Val, and Arg at P2′ (Fig. 2D). Hydrophobic residues were generally favored at P4 and P3′, whereas Pro and Arg were enriched at P4′. This substrate signature closely resembled that of the human aspartyl proteases cathepsin D and cathepsin E, which we previously characterized using the same MSP-MS library (Ivry et al., 2017).

Based on this similarity, we evaluated a commercially available cathepsin D/E fluorogenic substrate containing the peptide sequence GKPILFFRL flanked by 4-methylcoumarin amide (mca) and 2,4-dinitrophenyl (dnp) (Fig. 2E). This substrate incorporates several residues enriched in the MSP-MS-derived substrate signature, including Ile at P3, Phe at P1 and P1′, and Arg at P2′. Incubation with conditioned media acidified to pH 2.5 resulted in a rapid increase in fluorescence over 15 min, whereas cleavage was completely abolished by pepstatin (Fig. 2F). These results demonstrate that GKPILFFRL functions as a sensitive fluorescent reporter of *C. posadasii* aspartyl protease activity.

To further characterize this activity, cleavage of GKPILFFRL was measured across a pH range of 2.2–9.5. Maximal activity was observed between pH 3.0 and 3.8, with greater than 70% reduction in activity below pH 2.5 and above pH 5.0 (Fig. 2G). Although substantially reduced, substrate cleavage remained detectable at pH 7.5, corresponding to approximately 15% of maximal activity. Because fungal aspartyl proteases are not typically active across such a broad pH range, we tested whether cleavage at neutral pH remained pepstatin-sensitive. Pepstatin significantly inhibited substrate cleavage at pH 3.5 and 5.5 but had no effect at pH 7.5 (Fig. 2H), indicating that the residual activity observed at neutral pH is mediated by a distinct protease class.

These findings identify a dominant pepstatin-sensitive aspartyl protease activity in *C. posadasii* conditioned media with an acidic pH optimum between 3.0 and 3.8. The detection of pepstatin-insensitive cleavage at neutral pH further suggests the presence of additional secreted proteases with distinct biochemical properties.

### Neutral pH profiling reveals extensive aminopeptidase activity in *C. posadasii* conditioned media

To characterize protease activity at neutral pH, the same conditioned media was analyzed by MSP-MS at pH 7.4. Following a 1-hour incubation with the peptide library, 246 cleavage sites were detected, representing 8.3% of all possible peptide bonds within the library (2,964 total). In contrast to the limited activity observed at pH 2.5, only 7 cleavage sites were shared between the two conditions (Fig. 3A), indicating that proteolytic activity at neutral pH is substantially more extensive and largely distinct from that observed under acidic conditions.

**Fig. 3:**
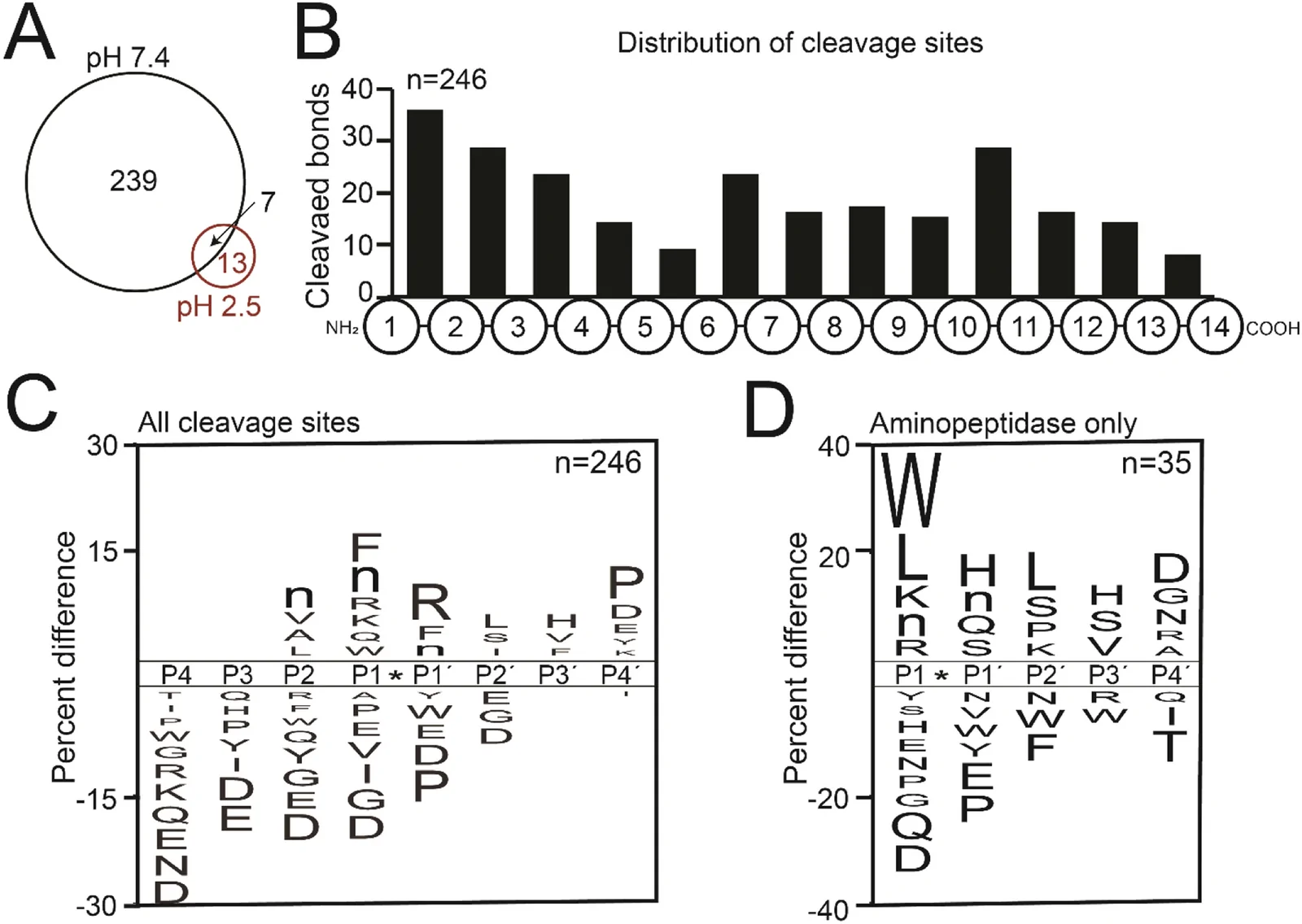
Substrate specificity profiling of *C. posadasii* secreted proteases at pH 7.4. (**A**) Venn diagram illustrating the overlap and differences in cleaved peptides observed at pH 2.5 and 7.4. (**B**) Distribution of cleavage sites within the 14-mer peptide library at pH 7.4. (**C**) Substrate signature (P4 to P4′) of the proteases active at pH 7.4. (**D**) Substrate signature (P1 to P4′) of the monoaminopeptidases active at pH 7.4. All assays were performed in quadruplicate technical replicates.

Analysis of cleavage site distribution revealed proteolysis throughout the peptide library, with the highest frequency occurring between residues 1 and 2 (Fig. 3B). Notably, removal of the N-terminal amino acid was observed in 35 of the 228 peptides, suggesting substantial monoaminopeptidase activity in the conditioned media.

Consistent with this observation, substrate signature analysis identified strong enrichment of norleucine (Nle) at P2, Phe and Nle at P1, Arg at P1′, and Pro at P4′ (Fig. 3C). In contrast, no amino acid enrichment was detected at the P4 or P3 positions. Because cleavage near the amino terminus precludes interactions with the S4 and S3 binding pockets, the absence of P4 and P3 preferences is a hallmark of aminopeptidase activity (Lapek et al., 2019; Jiang et al., 2021).

To further define aminopeptidase substrate specificity, the 35 cleavage sites occurring between residues 1 and 2 were analyzed separately. Comparison with all 228 available N-terminal sites revealed preferential cleavage of peptides containing Trp, Leu, Lys, Nle, and Arg at P1, whereas peptides containing Asp at P1 were rarely cleaved (Fig. 3D). Together, these findings identify aminopeptidases as a major component of the neutral pH proteolytic activity secreted by *C. posadasii*.

To validate the aminopeptidase specificities identified by MSP-MS, a panel of fluorogenic amino acid substrates was assembled in which individual amino acids were coupled to 7-amino-4-methylcoumarin (amc). Incubation with *C. posadasii* conditioned media revealed efficient cleavage of Lys-amc, followed by Leu-amc, Nle-amc, Trp-amc, and Arg-amc (Fig. 4A). In contrast, Asp-amc and Ser-amc, which were not enriched at P1 in the MSP-MS analysis, showed no detectable cleavage. Met-amc was also efficiently hydrolyzed. Although methionine was not included in the MSP-MS library because of its susceptibility to oxidation, its isostere Nle was strongly enriched, suggesting that the responsible aminopeptidase(s) readily accommodate both residues. Lys-amc and Met-amc exhibited similar pH-activity profiles, with maximal activity near pH 7.0 and greater than 90% reduction in activity at pH 6.0 and 9.0 (Fig. 4B), consistent with the presence of a neutral pH aminopeptidase activity.

**Fig. 4:**
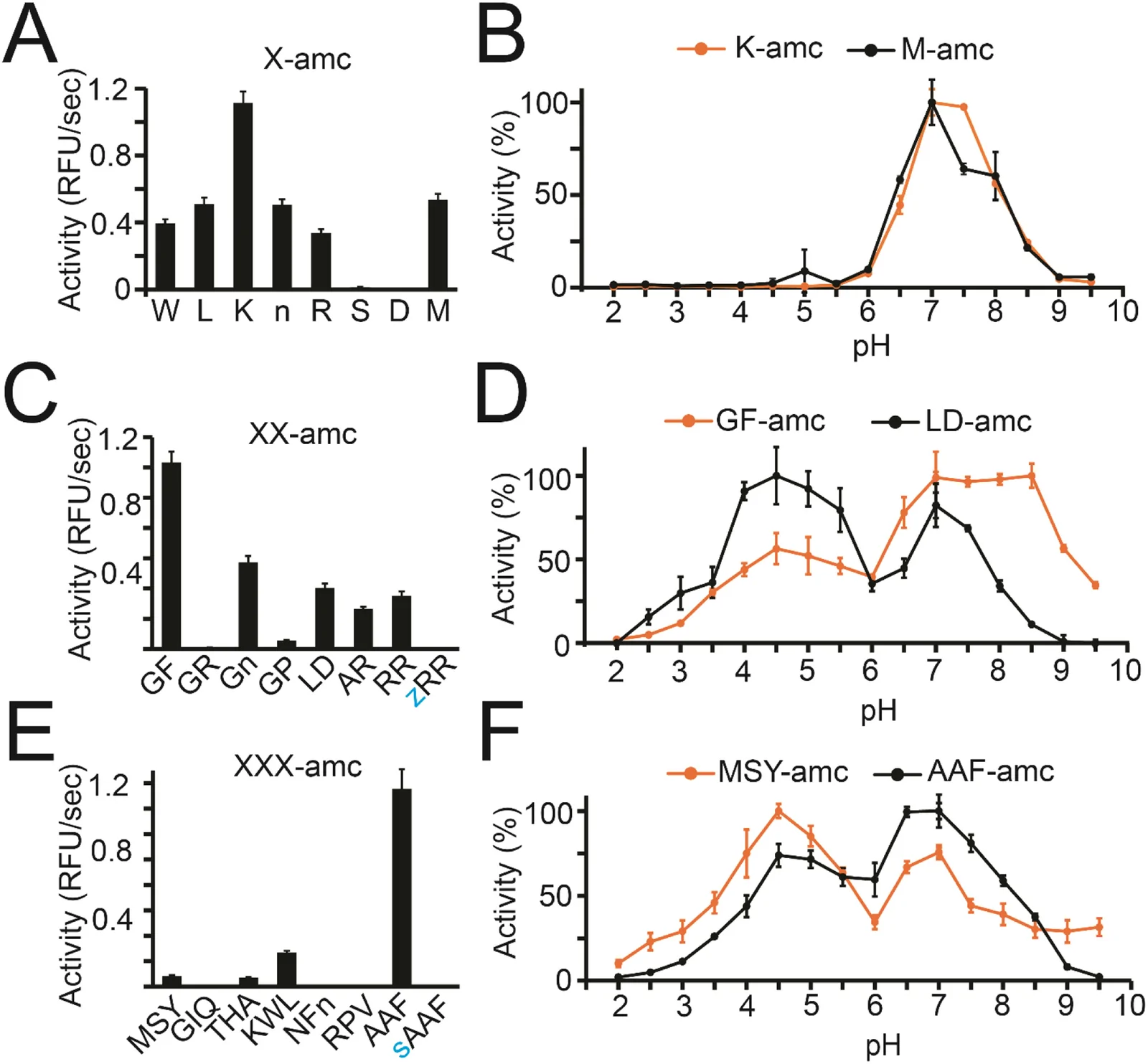
Validation of aminopeptidase activity with fluorogenic reporter substrates. (**A**) Hydrolysis of single amino acid-amc substrates (X-amc) by *C. posadasii* conditioned media. The increase in fluorescence was measured over time and expressed as RFU/s. (**B**) Substrate cleavage rates for X-amc substrates across a range of pH values. Cleavage rates were normalized to the highest observed activity. (**C**) Hydrolysis of dipeptide-amc substrates (XX-amc) by *C. posadasii* conditioned media. (**D**) Substrate cleavage rates for two XX-amc substrates across a range of pH values. (**E**) Hydrolysis of tripeptide-amc substrates (XXX-amc) by *C. posadasii* conditioned media. (**F**) Substrate cleavage rates for two XXX-amc substrates across a range of pH values. All assays were performed in three technical replicates.

Analysis of the MSP-MS cleavage distribution further revealed frequent cleavage between residues 2 and 3 (28 sites) and residues 3 and 4 (23 sites) (Fig. 3B), suggesting the presence of di- and triaminopeptidase activities in addition to monoaminopeptidases. To investigate this possibility, a panel of dipeptide-amc and tripeptide-amc substrates was screened. Four of the seven dipeptide substrates contained Gly at P2, a residue that was poorly tolerated by the monoaminopeptidases, thereby minimizing sequential cleavage. Among these substrates, Gly-Phe-amc was hydrolyzed most efficiently, followed by Gly-Nle-amc and Gly-Pro-amc, whereas Gly-Arg-amc was not cleaved (Fig. 4C). Gly-Phe-amc exhibited maximal activity between pH 7.0 and 8.5, with substantially reduced activity at pH 6.0 and 9.5 (Fig. 4D). Interestingly, activity increased again below pH 6.0, producing a second activity peak centered near pH 4.5. This biphasic profile is consistent with the presence of multiple peptidase activities capable of hydrolyzing the substrate.

Additional evidence for diaminopeptidase activity was obtained using Leu-Asp-amc. Because Asp-amc was not cleaved by the monoaminopeptidases, hydrolysis of Leu-Asp-amc would require removal of the N-terminal dipeptide. This substrate was cleaved by proteases in conditioned media (Fig. 4C) and exhibited activity maxima near pH 4.5 and 7.0 (Fig. 4D), again suggesting the presence of multiple diaminopeptidase activities. Ala-Arg-amc and Arg-Arg-amc were also hydrolyzed; however, blocking the N-terminus of Arg-Arg-amc with a benzyloxycarbonyl (Z) group completely abolished cleavage. Because a free N-terminus is required for aminopeptidase recognition, these findings confirm that the observed activity is mediated by diaminopeptidases.

To evaluate triaminopeptidase activity, seven tripeptide substrates were screened. Four substrates were cleaved, with Ala-Ala-Phe-amc exhibiting the highest activity (Fig. 4E). Cleavage was completely abolished by N-terminal succinylation, confirming dependence on a free amino terminus. The corresponding activity displayed a pH optimum near 7.0 (Fig. 4F). In contrast, Met-Ser-Tyr-amc was cleaved more efficiently at pH 4.5 than at pH 7.0, indicating the presence of an additional triaminopeptidase activity with a more acidic pH optimum. Together, these data demonstrate that *C. posadasii* secretes multiple aminopeptidase activities with distinct substrate preferences and pH profiles.

In addition to aminopeptidase-mediated cleavage, MSP-MS identified 160 cleavage events between residues 4 and 14 of the peptide substrates, suggesting the presence of endopeptidases and, near the C-terminus, potential carboxypeptidases. To selectively monitor these activities, we screened a panel of internally quenched fluorogenic substrates containing an N-terminal mca group and a C-terminal Lys(dnp)-OH quencher. As the mca group blocks the N-terminus, it prevents aminopeptidase-mediated cleavage of these substrates, thereby restricting hydrolysis to endopeptidases and carboxypeptidases. All substrates tested were cleaved by conditioned media, with mca-FnKRR-K(dnp)-OH exhibiting the highest activity at pH 7.4 (Fig. 5A). This substrate displayed maximal activity near pH 6.2, with greater than 75% reduction at pH 5.5 and 9.0 (Fig. 5B). In contrast, the cathepsin D/E substrate GKPILFFRL exhibited an acidic pH optimum, whereas mca-RPPGFSAF-K(dnp)-OH was cleaved most efficiently near pH 5.5. These distinct pH profiles suggest the presence of multiple endopeptidase activities with different biochemical properties.

**Fig. 5:**
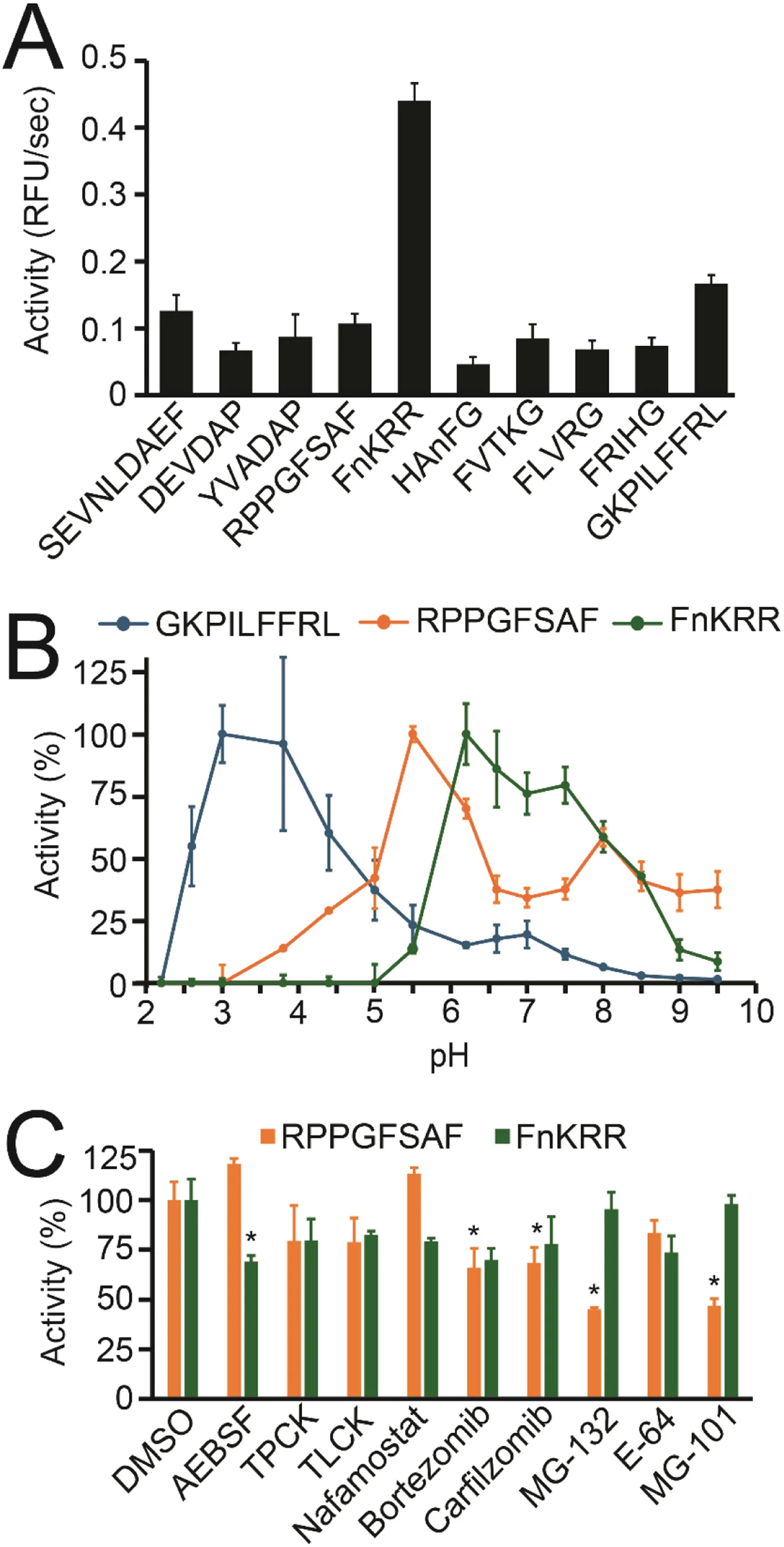
Detection of endopeptidases and carboxypeptidase activity using internally quenched fluorescent substrates. (A) Hydrolysis of internally quenched fluorescent substrates by proteases in *C. posadasii* conditioned media. The increase in fluorescence was measured over time and expressed as RFU/s. (**B**) Substrate cleavage rates across a range of pH values. Cleavage rates were normalized to the highest observed activity. (**C**) Screening of broad-spectrum protease inhibitors to identify the protease class responsible for substrate cleavage.

To further classify these activities, conditioned media was pre-incubated with protease inhibitors targeting serine, threonine/proteasome, and cysteine proteases prior to substrate cleavage assays at pH 7.4 (Table 2). Hydrolysis of RPPGFSAF was significantly inhibited by threonine protease inhibitors and the cysteine protease inhibitor MG-101, whereas cleavage of FnKRR was significantly reduced only by AEBSF. These findings indicate that distinct enzymes are responsible for cleavage of the two substrates and are consistent with the presence of both serine- and threonine-dependent endopeptidase activities in *C. posadasii* conditioned media.

In summary, these findings reveal a complex secreted protease repertoire in *C. posadasii* consisting of aspartyl protease activity at acidic pH, multiple aminopeptidase activities, diaminopeptidases and triaminopeptidases with distinct pH optima, and at least two endopeptidase activities corresponding to serine- and threonine-dependent proteases.

## Discussion

This study provides an in-depth activity-based profile of protease activities present in conditioned media from *C. posadasii* NR-166 mycelial cultures. Using multiplex substrate profiling by mass spectrometry (MSP-MS) and complementary fluorogenic substrate assays, we identified a diverse repertoire of extracellular proteolytic activities, including aspartyl protease, aminopeptidase, diaminopeptidase, triaminopeptidase, and serine- and threonine-dependent endopeptidase activities. These activities exhibited distinct substrate preferences, inhibitor sensitivities, and pH optima, demonstrating substantial biochemical diversity in the extracellular protease activity of this strain under the growth conditions examined. Several of these activities could be monitored using fluorogenic reporter substrates, establishing an activity-based framework for future studies aimed at defining the responsible enzymes, their biological roles during infection, and their potential utility in protease-targeted diagnostic approaches for Valley fever.

Current diagnostic approaches for Valley fever rely primarily on serological testing, culture, and imaging, each of which has important limitations, particularly during early infection. These limitations have motivated the development of complementary pathogen-directed approaches, including assays targeting fungal metabolites and nucleic acids (Chaturvedi et al., 2021; Higgins Keppler et al., 2021). Activity-based protease detection offers a distinct strategy because proteases can generate amplified signal through catalytic substrate turnover and can be monitored using relatively simple fluorogenic or colorimetric reporters. We and others have applied this general approach in several disease settings, including SARS-CoV-2 and norovirus infection, periodontitis caused by *Porphyromonas gingivalis*, pancreatic cyst neoplasia, and *C. albicans* biofilm formation (Ivry et al., 2017; Winter et al., 2016; Jin et al., 2023; Jin et al., 2022; Jin et al., 2022; Yeung et al., 2023; Retout et al., 2023). Although the present study does not establish diagnostic performance in clinical specimens, the identification of reproducible *C. posadasii* protease activities and corresponding reporter substrates provides a starting point for evaluating whether such activities can ultimately be exploited as pathogen-associated biomarkers.

Previous studies of *Coccidioides* proteases have focused primarily on individual enzymes implicated in extracellular protein degradation or virulence. *C. immitis* secreted at least two serine protease activities with distinct pH optima and substrate preferences, as well as an elastinolytic metalloprotease capable of degrading host extracellular matrix components (Lopes et al., 2008; Yuan and Cole, 1987; Resnick et al., 1987). More recently, transcriptomic profiling of *C. posadasii* strain Silveira during spherule development identified enrichment of multiple protease-encoding genes, including the metalloproteases Mep1, Mep7, and Kex2 and several serine proteases belonging to the S8, S9, S10, and S53 families (Homer et al., 2025). Among the best-characterized *C. posadasii* proteases are Pep1 and Mep1. Pep1 is an immunogenic aspartyl protease that elicits protective immunity when used as a recombinant vaccine antigen in murine infection models (Tarcha et al., 2006), whereas Mep1 is released during endosporulation and degrades an immunodominant surface antigen, thereby reducing immune recognition of newly released endospores (Hung et al., 2005; Donovan et al., 2019). Taken together, these studies demonstrate that *Coccidioides* produces multiple proteases with potential roles in host interaction and virulence, but they do not define the broader spectrum of extracellular proteolytic activities present under a given growth condition. Our activity-based profiling complements these prior studies by directly measuring protease function in conditioned media.

A major finding of the present study was the identification of a dominant pepstatin-sensitive protease activity under acidic conditions. MSP-MS profiling revealed a substrate preference resembling those of the human aspartyl proteases cathepsin D and cathepsin E, and a commercially available cathepsin D/E fluorogenic substrate was efficiently cleaved by *C. posadasii* conditioned media. Complete inhibition of substrate cleavage by pepstatin at acidic pH further supported classification of this activity as an aspartyl protease. Among the candidate enzymes previously identified in *Coccidioides* proteomic datasets, Pep1 is a plausible contributor to this activity. Pep1 shares greater sequence identity with cathepsin D and cathepsin E than does Pep2 and has previously been shown to be secreted and enzymatically active (Tarcha et al., 2006). These observations are consistent with Pep1 contributing to the cathepsin D/E-like activity detected here; however, sequence similarity and inhibitor sensitivity alone are insufficient to assign the activity to a specific enzyme. Definitive identification will require direct biochemical characterization or genetic validation. Regardless of the responsible enzyme, the efficient cleavage of the cathepsin D/E reporter demonstrates that acidic *C. posadasii* aspartyl protease activity can be monitored using a simple fluorogenic assay.

An important consideration for future diagnostic development is the potential contribution of host proteases to reporter substrate cleavage. Cathepsins D and E are primarily localized to intracellular endosomal and lysosomal compartments, but extracellular activity has been reported in several pathological settings, including cancer and inflammatory disease (Benes et al., 2008; O’Donoghue et al., 2016). Thus, host-derived protease activity could potentially contribute to background cleavage in clinical specimens. Future studies should therefore evaluate the cathepsin D/E reporter in healthy control samples, disease-control specimens, and samples from patients with coccidioidomycosis to determine the extent of host-derived background activity and whether substrate optimization can improve selectivity for *Coccidioides*-associated protease activity.

Beyond the acidic aspartyl protease activity, MSP-MS profiling revealed extensive aminopeptidase activity at neutral pH. Cleavage-site distributions and fluorogenic substrate assays demonstrated that *C. posadasii* conditioned media can remove one, two, or three amino acids from peptide N termini, consistent with mono-, di-, and triaminopeptidase activities. Efficient hydrolysis of Met-amc further indicated methionine aminopeptidase-like activity, although the responsible enzyme was not identified. The distinct pH optima observed for several dipeptide and tripeptide substrates suggest that multiple aminopeptidase activities with different biochemical properties are present in the conditioned media. Notably, several activities were retained or enhanced under moderately acidic conditions around pH 4.5. Because many host aminopeptidases exhibit maximal activity near neutral pH, these acidic activities may prove useful for future assay development by providing conditions that reduce background cleavage from host enzymes while preserving fungal protease activity.

The present study also identified multiple endopeptidase activities in *C. posadasii* conditioned media. Internally quenched fluorogenic substrates revealed activities with distinct substrate preferences and pH profiles, indicating that several proteolytic activities contribute to extracellular peptide cleavage. Inhibitor profiling further suggested the presence of both serine- and threonine/proteasome-like activities. Cleavage of FnKRR was reduced by AEBSF, consistent with a serine protease contribution and with prior reports of secreted serine proteases in *Coccidioides* species (Lopes et al., 2008; Yuan and Cole, 1987). In contrast, cleavage of RPPGFSAF was reduced by the proteasome inhibitors bortezomib, carfilzomib, and MG-132, supporting the presence of a threonine/proteasome-like activity. MG-101 also reduced RPPGFSAF cleavage, whereas E-64 had no effect. Because MG-101 can inhibit proteasome activity in fungi (Lee and Goldberg, 1996), this pattern is more consistent with inhibition of a proteasome-like activity than with a cysteine protease. However, inhibitor sensitivity alone does not identify the responsible enzymes, and definitive assignment will require direct biochemical or genetic validation.

A key strength of MSP-MS is that it directly measures proteolytic activity rather than inferring enzyme function from transcript or protein abundance. Detection of a protease by transcriptomics or proteomics does not establish whether the enzyme is catalytically active under a given condition, whereas MSP-MS provides a functional readout of substrate cleavage across a diverse peptide library. This approach has previously been used to identify disease-associated protease activities in *Pseudogymnoascus destructans* (O’Donoghue et al., 2015), distinguish planktonic and biofilm-associated proteolytic profiles in *C. albicans* (Winter et al., 2016), and identify protease biomarkers in pancreatic cyst fluid (Ivry et al., 2017). Our findings extend this activity-based strategy to *Coccidioides* and demonstrate its utility for defining extracellular proteolytic activities that may not be apparent from expression or abundance measurements alone.

Several limitations should be considered when interpreting these results. First, protease activity was characterized using cell-free conditioned media derived from predominantly mycelial cultures grown under laboratory conditions. Because our analyses were performed using conditioned media, we cannot exclude the possibility that some of the detected protease activities derive from proteins released during cell turnover or lysis rather than active secretion. In addition, because *Coccidioides* undergoes a dramatic morphological transition during infection, the protease repertoire of parasitic spherules may differ substantially from that observed here. It will therefore be important to directly characterize extracellular protease activity from spherules using approaches similar to those employed in this study. Second, the molecular identities of several proteases responsible for the observed activities remain unresolved. Although inhibitor profiles, substrate preferences, and pH optima provide important clues regarding enzyme classification, definitive assignment will require biochemical purification, proteomic identification, or genetic approaches. Third, this study used the standard avirulent *C. posadasii* lab strain NR-166, which lacks three chitinase genes. Although it is not known whether this strain differs from virulent isolates in its extracellular protease profile, future studies should evaluate protease activity across multiple clinically relevant strains. Finally, the fluorogenic substrates described here have not yet been evaluated in infected animals or clinical specimens, where host-derived proteases and endogenous inhibitors may influence substrate cleavage and assay specificity.

Future studies should focus first on identifying the proteases responsible for the most promising activities and defining their substrate preferences in greater detail. Expanded substrate libraries incorporating non-natural amino acids could then be used to improve selectivity for *Coccidioides*-derived protease activity while minimizing cleavage by host enzymes, an approach that has proven useful for human, bacterial, and fungal proteases (Winter et al., 2016; Poreba et al., 2018; Rut et al., 2018; Lentz et al., 2016; Mahoney et al., 2024). Once optimized reporter substrates are available, their performance should be evaluated in spherule cultures, virulent *Coccidioides* strains, animal infection models, and ultimately clinical specimens. Alternative detection platforms, including surface-enhanced Raman scattering (SERS), could subsequently be explored to improve analytical sensitivity and facilitate implementation in clinical laboratory settings. These studies will be necessary to determine whether the protease activities identified here can be developed into reliable biomarkers of Valley fever.

In summary, this study provides an in-depth activity-based profile of extracellular protease activities present in conditioned media from *C. posadasii* NR-166 mycelial cultures. We identified multiple proteolytic activities with distinct substrate preferences, pH optima, and inhibitor sensitivities and developed fluorogenic reporter substrates capable of monitoring several of these activities in real time. These findings complement recent gene-expression studies indicating that proteases may contribute to *Coccidioides* adaptation and virulence (Homer et al., 2025) and establish a framework for future studies aimed at identifying the responsible enzymes, defining their biological functions during infection, and evaluating their potential utility as activity-based biomarkers of Valley fever.

**Supplementary File 1**. Excel sheet consisting of processed mass spectrometry data for each MSP-MS experiment.

## Declaration of competing interest

The authors declare the following financial interests/personal relationships which may be considered as potential competing interests: Anthony J. O’Donoghue reports financial support was provided by the National Institutes of Health (NIH). Clarissa Nobile reports financial support was provided by the NIH. Clarissa Nobile is a cofounder of BioSynesis, Inc., a company developing diagnostics and therapeutics for biofilm infections. All other authors declare that they have no known competing financial interests or personal relationships that could have appeared to influence the work reported in this paper.
